# Supplementary material for: Non-telecentric two-photon microscopy for 3D random access mesoscale imaging
Source: Nat Commun. 2022 Jan 27;13:544. doi: 10.1038/s41467-022-28192-0 (PMC8795402; doi:10.1038/s41467-022-28192-0)
Supplement: Supplementary file 1 — Supplementary Information [file 41467_2022_28192_MOESM1_ESM.pdf]

SUPPLEMENTARY MATERIALS

Non-Telecentric two-photon microscopy for 3D random access mesoscale imaging

Janiak et al.

Optical Aberrations and their possible impact in nTC. In general, optical aberrations can be divided into two groups, chromatic and monochromatic. In 2P microscopy, chromatic aberrations generally do not apply because the excitation laser is essentially monochromatic and collection does not depend on image focus. Nevertheless, monochromatic aberrations still depend on wavelength. Generally, shorter wavelengths yield greater aberration. As a consequence, at a given focal depth, achromatic aberrations tend to be approximately half as strong in 2P microscopy compared to 1P microscopy[47].

Next, in 2P imaging monochromatic aberrations are mainly related to the sample rather than the imaging system itself. Here, low-order aberrations are introduced by inevitable small differences in the refractive index between the sample, the immersion fluid (usually water) and the objective[49], alongside typically higher-order aberrations due to refractive index inhomogeneities within biological samples (e.g. blood vessels, dura, neuropil, cell bodies)[46,74]. Generally, aberrations introduced by within-tissue inhomogeneities tend to be smaller than those introduced by the immersion medium[49].

The specific design of our nTC excitation path primarily impact low order monochromatic aberrations. These are inevitably dominated by depth-dependent first order spherical aberrations[44–49]. Spherical aberration means that peripheral and axial rays do not converge to a point, thereby broadening the focal excitation spot (related to PSF). In addition, spherical aberrations also cause a lateral focal shift which depends on the distance to the FOV centre[45]. In 1P widefield microscopy, these are the primary limit for achievable image resolution[75], while in 1P confocal microscopy they lead to a reduction in signal power[76]. Similarly under 2P, excitation (which is quadratically related to photon density) is diminished at increasing depth due to spherical aberrations[48].

There are a few options that can be explored for reducing aberrations in 2P microscopy. For example, the use of optical clearing agents would help, however to date there is no protocol available for *in-vivo* imaging[77]. Moreover, most (but not all) aberrations can, in principle, be pre-corrected by adaptive optics including by way of wavefront shaping using a spatial light modulator or deformable mirror[56,78–80]. Important calibration of adaptive optics can be achieved with a wavefront sensor[78,81,82] or even without it[46,49,83–85]. The use of a sensor usually allows for faster and higher precision calibration for a specific sample and depth, however this requires an endogenous structure with known shape (often called a “guide star”)[78] which may require surgery[86]. Notably, adaptive optics can be used to correct aberrations related to the sample itself, including surface shape, angle or inhomogeneity[46,74]. In principle, our nTC approach is compatible if any of the above approaches to reducing aberrations. This would also allow imaging deeper, potentially further increase the maximal achievable z-shift through the ETL, and address imaging field curvature inevitable in large FOV microscopy[74].

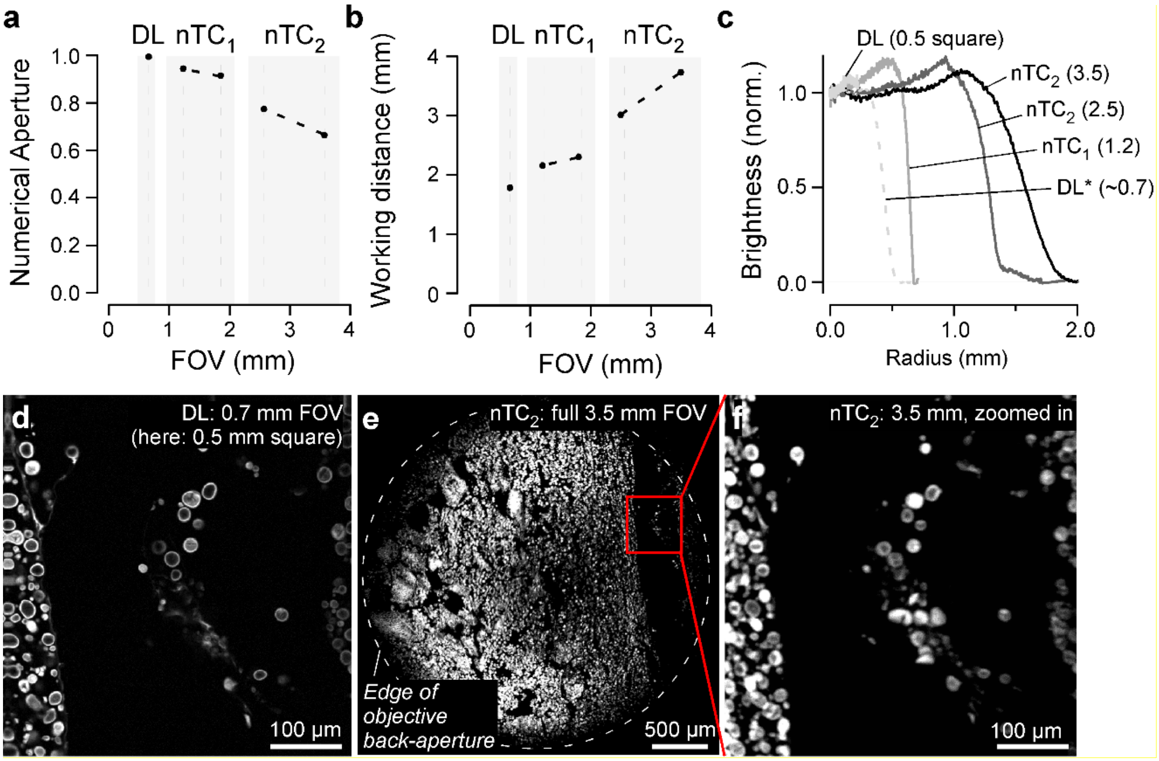

***Figure S1 | related to Figure 1. nTC optical performance. a**, Numerical Aperture (N.A.) calculated for the different optical configurations and **b**, measured working distance. **c**, Image brightness of the different configurations quantified from scans of a block of agarose with fluorescein. The two traces for the DL configuration denote the brightness over the ‘native’ 0.5 mm square FOV of the Sutter MOM equipped with the used Zeiss x20 Objective (DL), and of the total usable FOV of the objective ‘in isolation’ (~0.7 mm, DL*), here measured by artificially offsetting one of the scan mirrors such that the scanning laser beam could go beyond the edge of the*

objective back-aperture. ***d-f****, fluorescence test-slide imaged under DL-configuration at full 0.5 square mm FOV (d), at nTC2 3.5 mm, at full FOV (e) and at nTC2 3.5 mm zoomed in to the same sample position as in (d). Individual sphere-like structures (~10 µm in diameter, i.e. similar or smaller than most somata in the adult mouse brain, e.g. Ref[87], remain readily* discernible. *Data leading to a-c in Source Data file.*

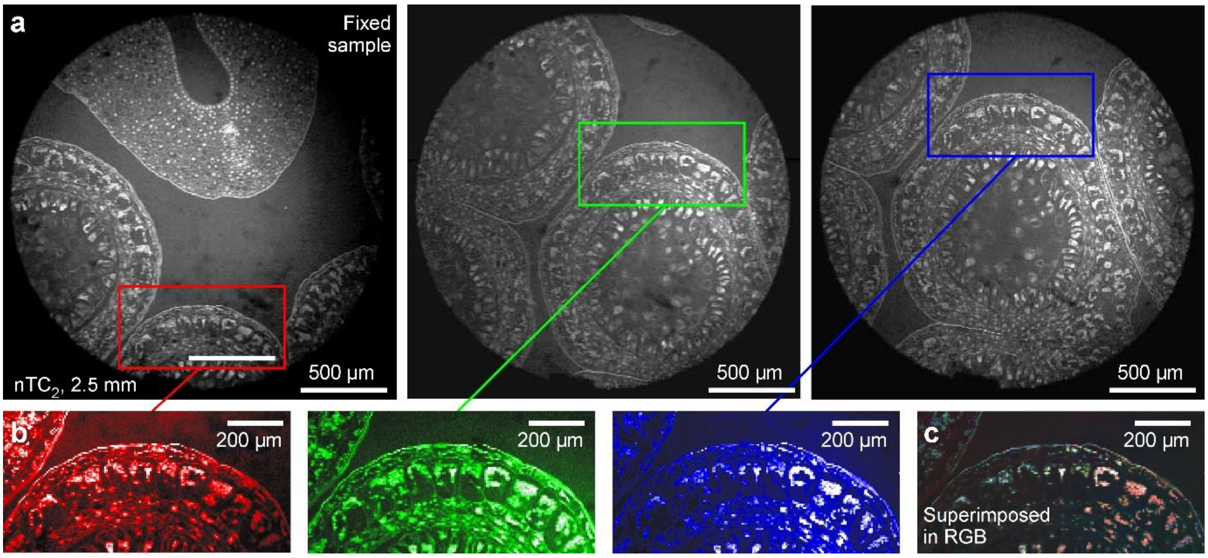

Figure S2 | related to Figure 2. No obvious image distortion across full field of view. a*, example full field of view scans of a fixed lily pollen sample under nTC2 2.5 mm configuration at three different xy-stage translations. In each case the image boundary is determined by the objective back aperture.* ***b****, image crops of the same image structure from (a) as indicated, here false colour coded in RGB-channels, and* ***c****, superposition of all three image crops. Note that the three image-crops superimpose well, meaning that any possible image distortion across the full field of view, if present, is very small.*

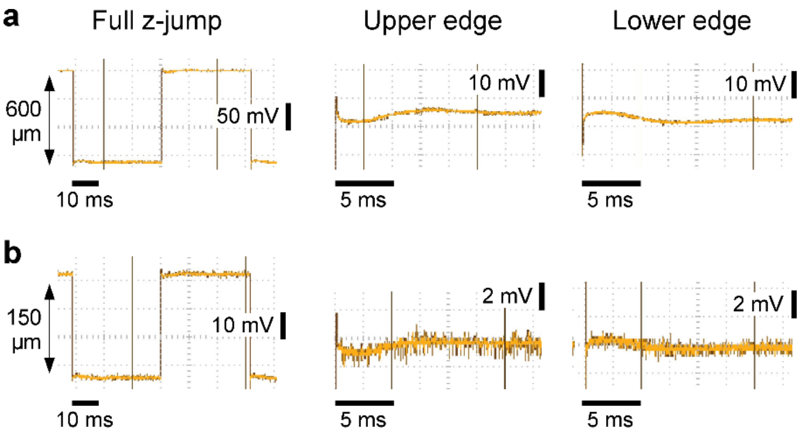

Figure S3 | related to Figure 4. ETL settling time. *Voltage on the electrically tunable lens (ETL) recorded on an oscilloscope through a resistor as a readout of current curvature. In response to current step commands that resulted in 600 (top) and 150 (bottom) µm axial focus jumps, the lens oscillated with <5% maximal jump amplitude following an initial sub-millisecond transient. This oscillation reliably settled beyond detection limit within <10 ms (600 µm jump). For smaller jumps it settled substantially earlier.*

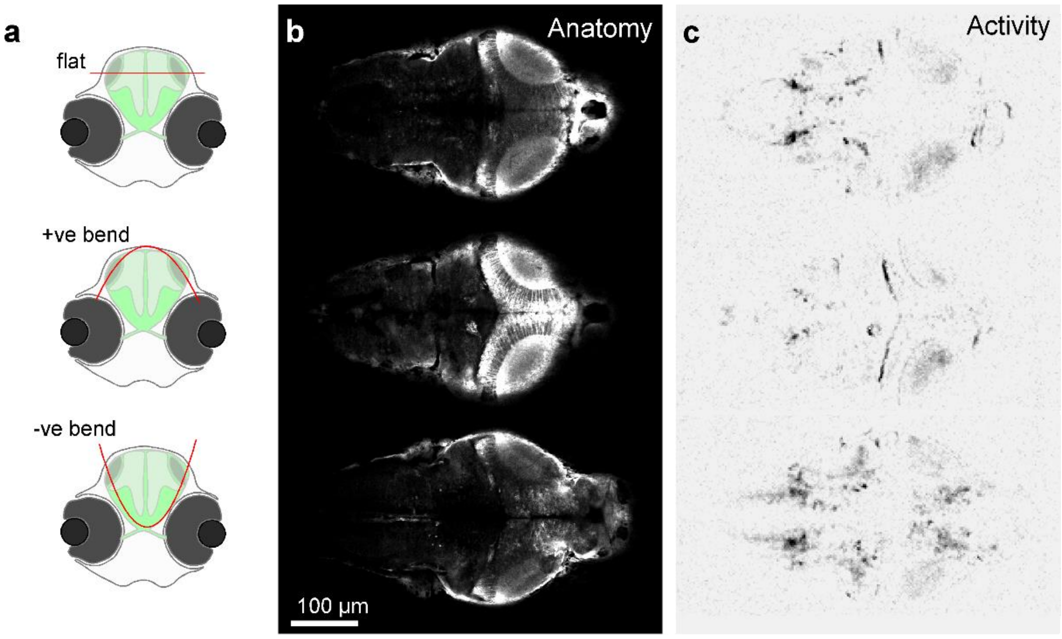

***Figure S4 | related to Figure 7. Staggered plane bending. a**, Schematic of HuC:GCaMP6f larval zebrafish shown from front, with scan-planes indicated. b-c, three times 170x340 px (1.96 Hz volume rate) staggered bent-planes used to quasi-simultaneously capture the brain at three different orientations as indicated, with mean image (b), and activity-correlation during spontaneous activity (c, cf. Fig. 3c).*

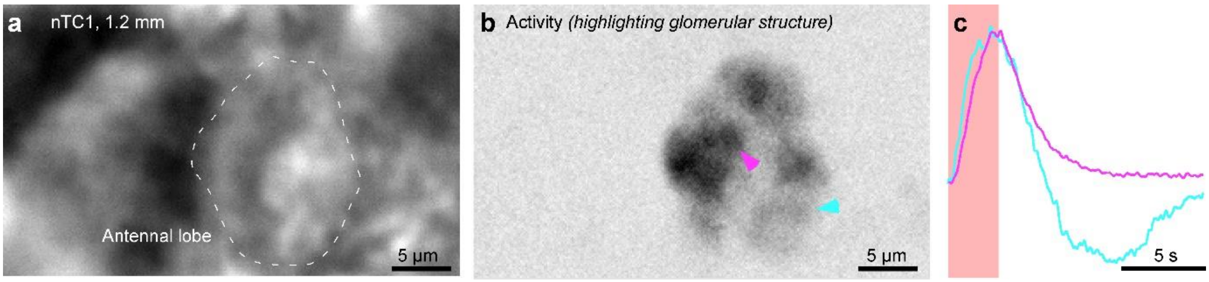

Figure S5 | related to Figure 10. Optogenetic activation of Drosophila L1 antennal lobe. *As Fig. 9, a second Drosophila L1 sample scanned using nTC$_1$ (1.2 mm configuration) with a single plane (170x340 px, 5.88 Hz) and zoomed-in to the antennal lobe to ensure spatial oversampling.* ***a-c****, average-projection (a), activity-correlation (b, cf. Fig. 2e).) and example average time-traces from two glomeruli as indicated (c).*
