# Supplementary material for: Non-telecentric two-photon microscopy for 3D random access mesoscale imaging
Source: Nat Commun. 2022 Jan 27;13:544. doi: 10.1038/s41467-022-28192-0 (PMC8795402; doi:10.1038/s41467-022-28192-0)
Supplement: Supplementary file 3 — Description of Additional Supplementary Files [file 41467_2022_28192_MOESM3_ESM.pdf]

Description of Additional Supplementary Files

File Name: Supplementary Movie 1
Description: ***related to Figure 1 | Z-stack through three larval zebrafish**. nTC_2 z-stack (3.5 mm FOV) of three larval zebrafish as in Fig. 1e, 1,024x1,024 px (0.5 Hz per plane), 1 µm z-steps.*

File Name: Supplementary Movie 2
Description: ***related to Figure 2 | Scan-profiles.** Direct visualisation of scan-profiles from the side, visualised with a camera (cf. Fig. 2a). Shown, in sequence, are: DL full FOV, nTC_1 1.2 mm configuration full FOV, nTC_2 2.5 mm configuration full FOV, DL individual PSFs, nTC_2 2.5 mm configuration individual PSFs. All: x4 real time. Note, the first three instances are filmed continuously to illustrate how quickly optical configurations can be switched. Lens switching was done by hand.*

File Name: Supplementary Movie 3
Description: ***related to Figure 3 | Comparison of different optical configurations on same sample.** UV-light evoked mean responses of sparely labelled neurons in upper spinal tract of larval zebrafish, imaged consecutively with all five configurations, as shown in Figure 3. From left to right: DL, nTC_1 1.2 mm, nTC_1, 1.8 mm, nTC_2 2.5 mm, nTC_2 3.5 mm. All x8 real time.*

File Name: Supplementary Movie 4
Description: ***related to Figure 4 | Scan-profiles during ETL shifts.** Direct visualisation of nTC_1 1.2 mm configuration scan-profiles from the side during different scan-programmes that use the ETL (cf. Fig. 2b). All: x4 real time.*

File Name: Supplementary Movie 5
Description: ***related to Figure 5 | Mesoscale imaging of 2 zebrafish brains at the same time.** Light-stimulus triggered average response movie from 2 larval zebrafish brains as shown in Fig. 5b. Looped 9 times (x10 real-time).*

File Name: Supplementary Movie 6
Description: ***related to Figure 7 | Half-pipe imaging of larval zebrafish brain.** As in Fig. 7d, nTC_1 anatomical scan (1.2 mm FOV) of larval zebrafish brain, with increasing z-curvatures applied. 512x1,024 px (1 Hz per plane). Note that planes 3 and 4 most closely follow natural brain curvature.*

File Name: Supplementary Movie 7
Description: ***related to Figure S7 | Half-pipe multiplane imaging of larval zebrafish brain.** As in Fig. S5, nTC_1 scan (1.2 mm FOV) of larval zebrafish brain, with three different z-curvatures applied: none (top) positive (middle) and negative (bottom). 512x1,024 px (1 Hz per plane). Looped 3 times.*

File Name: Supplementary Movie 8
Description: ***related to Figure 8 | Mesoscale imaging of mouse brain slice.*** *nTC_2 scan (3.5 mm FOV) of seizure-like activity mouse brain slice, in 2 parts. First, FOV as shown in Fig. 8d (1,024x1,024 px, 0.5 Hz), and second as shown in Fig. 8h,i (2 times 128x256 px, 3.91 Hz each). Looped 3 times.*

File Name: Supplementary Movie 9
Description: ***related to Figure 9 | Mesoscale imaging of mouse cortex in vivo.*** *As in Fig. 9d, nTC_1 scan (1.5 mm FOV) of spontaneous activity in mouse somatosensory cortex at 1,024x1,024 px (0.5 Hz). Looped 3 times.*

File Name: Supplementary Movie 10
Description: ***related to Figure 10 | Multiplane optogenetics in Drosophila larva.*** *As in Fig. 10g,h, nTC_1 scan (1.2 mm FOV) of L1 larval Drosophila brain, with six planes scanned at 170x340 px, 0.98 Hz volume rate during optogenetic activation of CsChrimson in olfactory sensory neurons. Average of 10 stimulus repeats. (left: fluorescence average, right, background subtracted and false colour-coded). Looped 6 times.*
