# Supplementary material for: Non-telecentric two-photon microscopy for 3D random access mesoscale imaging
Source: Nat Commun. 2022 Jan 27;13:544. doi: 10.1038/s41467-022-28192-0 (PMC8795402; doi:10.1038/s41467-022-28192-0)
Supplement: Supplementary file 14 — Reporting Summary [file 41467_2022_28192_MOESM14_ESM.pdf]

nature research

Corresponding author(s): Janiak, Baden

Last updated by author(s): 2021 12 21

Reporting Summary

Nature Research wishes to improve the reproducibility of the work that we publish. This form provides structure for consistency and transparency in reporting. For further information on Nature Research policies, see our Editorial Policies and the Editorial Policy Checklist.

Statistics

For all statistical analyses, confirm that the following items are present in the figure legend, table legend, main text, or Methods section.

n/a	Confirmed	
☐	☒	The exact sample size (*n*) for each experimental group/condition, given as a discrete number and unit of measurement
☒	☐	A statement on whether measurements were taken from distinct samples or whether the same sample was measured repeatedly
☐	☒	The statistical test(s) used AND whether they are one- or two-sided<br>*Only common tests should be described solely by name; describe more complex techniques in the Methods section.*
☐	☒	A description of all covariates tested
☐	☒	A description of any assumptions or corrections, such as tests of normality and adjustment for multiple comparisons
☐	☒	A full description of the statistical parameters including central tendency (e.g. means) or other basic estimates (e.g. regression coefficient) AND variation (e.g. standard deviation) or associated estimates of uncertainty (e.g. confidence intervals)
☐	☒	For null hypothesis testing, the test statistic (e.g. *F*, *t*, *r*) with confidence intervals, effect sizes, degrees of freedom and *P* value noted<br>*Give P values as exact values whenever suitable.*
☒	☐	For Bayesian analysis, information on the choice of priors and Markov chain Monte Carlo settings
☒	☐	For hierarchical and complex designs, identification of the appropriate level for tests and full reporting of outcomes
☒	☐	Estimates of effect sizes (e.g. Cohen's *d*, Pearson's *r*), indicating how they were calculated

Our web collection on statistics for biologists contains articles on many of the points above.

Software and code

Policy information about availability of computer code

Data collection	Data was collected using the custom written 2P control software "ScanM" (By T Euler and M Mueller), running under Igor Pro 6 (Wavemetrics)
Data analysis	Analysis was performed using custom written scripts written in Igor Pro 6 (Wavemetrics) and Matlab 9.5 R2018b (Mathworks)

For manuscripts utilizing custom algorithms or software that are central to the research but not yet described in published literature, software must be made available to editors and reviewers. We strongly encourage code deposition in a community repository (e.g. GitHub). See the Nature Research guidelines for submitting code & software for further information.

Data

Policy information about availability of data

All manuscripts must include a data availability statement. This statement should provide the following information, where applicable:

- Accession codes, unique identifiers, or web links for publicly available datasets
- A list of figures that have associated raw data
- A description of any restrictions on data availability

All systematic data (e.g. PSF measurements) will be made freely available upon publication. All example data (e.g. example scans) will be provided upon request to t.baden@sussex.ac.uk.

Field-specific reporting

Life sciences study design

All studies must disclose on these points even when the disclosure is negative.

Sample size	No sample size calculations were performed. Sample sizes for PSF measurements were chosen to obtain an average and SD without correction for student's t-distribution, which was more than 10 in all cases.
Data exclusions	No data was excluded
Replication	All results have been verified by repeating experiments multiple times
Randomization	Randomisation was not required for this research because the goal was to show the functionality of our microscope rather than to investigate a biological process
Blinding	Blinding was not required for this research because the goal was to show the functionality of our microscope rather than to investigate a biological process

Reporting for specific materials, systems and methods

We require information from authors about some types of materials, experimental systems and methods used in many studies. Here, indicate whether each material, system or method listed is relevant to your study. If you are not sure if a list item applies to your research, read the appropriate section before selecting a response.

Materials & experimental systems

n/a	Involved in the study	
☒	☐	Antibodies
☒	☐	Eukaryotic cell lines
☒	☐	Palaeontology and archaeology
☐	☒	Animals and other organisms
☒	☐	Human research participants
☒	☐	Clinical data
☒	☐	Dual use research of concern

Methods

n/a	Involved in the study	
☒	☐	ChIP-seq
☒	☐	Flow cytometry
☒	☐	MRI-based neuroimaging

Animals and other organisms

Policy information about studies involving animals; ARRIVE guidelines recommended for reporting animal research

Laboratory animals	Zebrafish (Danio rerio, 6-7 dpf), Mice (Mus musculus, adult), fruit flies (Drosophila melanogaster, L1 and L3)
Wild animals	No wild animals were used in this study
Field-collected samples	No field-collected samples were used in this study
Ethics oversight	All animal experiments presented in this work were carried out in accordance with the UK Animal (Scientific Procedures) Act 1986 and institutional regulations at the University of Sussex. All procedures were carried out in accordance with institutional, national (UK Home Office PPL70/8400 (mice), PPL/PE08A2AD2 (zebrafish)) and international (EU directive 2010/63/EU) regulations for the care and use of animals in research.

Note that full information on the approval of the study protocol must also be provided in the manuscript.
